# Therapeutic challenges in managing *Stenotrophomonas maltophilia* bloodstream infection in a renal dysfunction patient: a case study and literature review

**DOI:** 10.3389/fcimb.2025.1694228

**Published:** 2025-10-24

**Authors:** Xiongxiong Fan, Feng Chen, Zhengshang Ruan, Lixia Li

**Affiliations:** ^1^ Department of Pharmacy, Xin Hua Hospital Affiliated to Shanghai Jiao Tong University School of Medicine, Shanghai, China; ^2^ Department of Clinical Pharmacy, Baoji Central Hospital, Baoji, Shaanxi, China; ^3^ Department of Clinical Laboratory, Xin Hua Hospital Affiliated to Shanghai Jiao Tong University School of Medicine, Shanghai, China; ^4^ Department of Intensive Care Unit, Xin Hua Hospital Affiliated to Shanghai Jiao Tong University School of Medicine, Shanghai, China

**Keywords:** *Stenotrophomonas maltophilia*, bloodstream infection, renal insufficiency, individualized treatment, antimicrobial combination testing

## Abstract

**Background:**

The overall infection rate of *Stenotrophomonas maltophilia* has markedly increased over the past two decades, with its bloodstream infections being associated with poor clinical outcomes and high mortality rates. In patients with concomitant renal insufficiency, the complexity of anti-infective therapy is further heightened due to limited antibiotic options and altered pharmacokinetics, highlighting the critical importance of individualized treatment strategies.

**Objective:**

This study aims to explore effective clinical treatment strategies for *S. maltophilia*-induced bloodstream infections in patients with renal insufficiency and to provide evidence-based support for optimizing antimicrobial decision-making.

**Methods:**

We present a case of *S. maltophilia* bloodstream infection occurring in a patient with concomitant renal insufficiency. The choice of antimicrobial agents, dosage modifications, and combination therapy were systematically analyzed based on results from antimicrobial combination testing and observed clinical response. A comprehensive review of relevant literature was also conducted.

**Results:**

Guided by the findings of antimicrobial combination testing, an individualized regimen consisting of ceftazidime-avibactam (CZA) and aztreonam (ATM) was implemented, with dose adjustments tailored according to the patient’s renal function. This approach led to the successful resolution of the infection. The literature review further supports that in patients with renal insufficiency, antimicrobial selection should be guided by considerations including nephrotoxic potential, spectrum of activity, and pharmacokinetic profiles. Combined susceptibility testing emerges as a valuable tool in tailoring effective therapeutic regimens.

**Conclusion:**

The combination of CZA and ATM, guided by antimicrobial susceptibility testing and adjusted according to individual patient characteristics, demonstrated both safety and efficacy in the treatment of *S. maltophilia* bloodstream infection.

## Introduction

*S. maltophilia* is a Gram-negative, nonfermentative opportunistic pathogen commonly found in both natural environments and healthcare settings. This organism primarily causes nosocomial infections in immunocompromised individuals (Brooke, 2012). Infections attributed to *S. maltophilia* can extend hospital stays by approximately 8 to 14 days (Fihman et al., 2012). Epidemiological studies indicate that the isolation rate of *S. maltophilia* in clinical mixed infection specimens ranges from 33% to 70% (Bostanghadiri et al., 2024), with pneumonia being the most frequently observed clinical manifestation. Besides respiratory tract infections, *S. maltophilia* can cause bloodstream infections, urinary tract infections, intra-abdominal infections, catheter-related infections, and implant-associated infections. Rarely, it may affect the cardiovascular system, bones, soft tissues, and central nervous system (Brooke, 2012).

This bacterium exhibits intrinsic resistance to various classes of antibiotics, including carbapenems, aminoglycosides, trimethoprim, and fosfomycin. Its resistance mechanisms encompass decreased cell membrane permeability, chromosomally encoded efflux pump systems, the production of β-lactamases, and the activity of aminoglycoside-modifying enzymes (Gil-Gil et al, 2020). According to the 2024 data from the China Antimicrobial Surveillance Network (CHINET, www.chinets.com) (Ding et al., 2024), the resistance rates of *S. maltophilia* to trimethoprim-sulfamethoxazole (TMP-SMX), tigecycline, and minocycline were found to be 6.5%, 5.9%, and 1.4%, respectively.

The clinical prognosis of *S. maltophilia* infections is closely associated with the infection site and underlying health status of the patient. Mortality rates for bloodstream infections range from 21% to 69%, while those for pulmonary infections can reach as high as 75% (Majumdar et al., 2022). Several independent risk factors for adverse outcomes have been identified, including admission to the ICU, septic shock, mechanical ventilation, placement of central venous catheter, neutropenia, hematologic malignancies, chronic kidney disease, inappropriate antimicrobial therapy, and recent antibiotic exposure (Kanchanasuwan et al., 2022; Huang et al, 2024). Among ICU patients, mortality rates are particularly elevated, often associated with complications such as septic shock and the necessity for mechanical ventilation. ICU-acquired infections are independently linked to an increased risk of mortality (Carbonell et al, 2024).

The 2024 guidelines from the Infectious Diseases Society of America (IDSA) (Tamma et al., 2024) recommend two treatment approaches for infections caused by *S. maltophilia*: (1) combination therapy using two agents chosen from cefiderocol, minocycline, TMP-SMX, or levofloxacin; (2) combination therapy involving ceftazidime-avibactam (CZA) plus aztreonam (ATM). This article presents and analyzes the therapeutic strategy employed in a case of *S. maltophilia*-induced bloodstream infection, with the aim of providing evidence-based guidance for the management of such infections.

## Case presentation

A 73-year-old male patient, weighing 65 kg, diagnosed with bilateral renal artery stenosis, underwent sequential stent placement in both renal arteries and the right lower limb artery during his hospitalization. His medical history included hypertension, type 2 diabetes mellitus, coronary atherosclerotic heart disease, a prior myocardial infarction with subsequent percutaneous coronary intervention (PCI), cardiac insufficiency, and chronic kidney disease. Following the surgical procedure, a decrease in oxygen saturation (SaO_2_) to approximately 80% necessitated his transfer to the surgical intensive care unit (SICU) for further management.

On SICU day 1, the patient’s vital signs and laboratory findings were recorded as follows: body temperature (T) at 36.3°C, blood pressure (BP) at 144/68 mmHg, respiratory rate (RR) at 27 breaths/min, and heart rate (HR) at 106 beats/min; a white blood cell count (WBC) of 73.99 × 10^9^/L, a neutrophil percentage (N%) of 94.6%, a platelet count (PLT) of 253 × 10^9^/L, a red blood cell count (RBC) of 2.26 × 10¹²/L, and a hemoglobin (Hb) level of 69 g/L. Additional findings included a C-reactive protein (CRP) level of 36 mg/L, blood urea nitrogen (BUN) of 10 mmol/L, serum creatinine (Cr) of 150 μmol/L, total bilirubin (TBIL) of 37.8 μmol/L, direct bilirubin (DBIL) of 0 μmol/L, alanine aminotransferase (ALT) of 23 U/L, aspartate aminotransferase (AST) of 39 U/L, and albumin (ALB) level of 27.8 g/L. On the 3rd day in the SICU, the patient underwent tracheal intubation due to a SaO_2_ level of 80%, despite receiving high-flow oxygen therapy at a flow rate of 45 L/min. From the 4th to the 19th day in the SICU, the patient developed postoperative complications, including pulmonary and wound infections. Microbial cultures identified mixed infections involving carbapenem-resistant *Acinetobacter baumannii* (CRAB), *Enterococcus faecalis*, *Klebsiella pneumoniae* (KP), *Pseudomonas aeruginosa* (PA), and *Enterobacter cloacae*. A sequence of antibiotic regimens was administered: piperacillin-tazobactam combined with linezolid, followed by cefoperazone-sulbactam plus vancomycin, and finally polymyxin B in conjunction with meropenem. The complete treatment regimens are summarized in **Table 1**.

**Table 1:** Antibiotics used for anti-infection treatment during the patient’s hospitalization.

Antimicrobial drugs	Administration and dosage	Administration time
Piperacillin-Tazobactam	4.5g q12h ivgtt	D1-D5
Linezolid	0.6g q12h po	D1-D9
Cefoperazone-Sulbactam	3g q12h ivgtt	D6-D17
Fluconazole	200mg qd ivgtt	D9-D27
Meropenem	1g q12h ivgtt	D18-D22
Polymyxin B	500,000 U q12h ivgtt	D18-D20
Minocycline	100mg qd po	D20-D22
Polymyxin B	1,000,000 U q12h ivgtt	D21-D23
Ceftazidime-Avibactam	1.25g q8h ivgtt	D23-D28
Aztreonam	1g q8h ivgtt	D23-D28
Polymyxin B	500,000 U q12h ivgtt	D24-D30
Ceftazidime-Avibactam	2.5g q8h ivgtt	D29-D34
Aztreonam	2g q8h ivgtt	D29-D34

ivgtt: intravenous guttae; po: oral.

**Figure 1:**
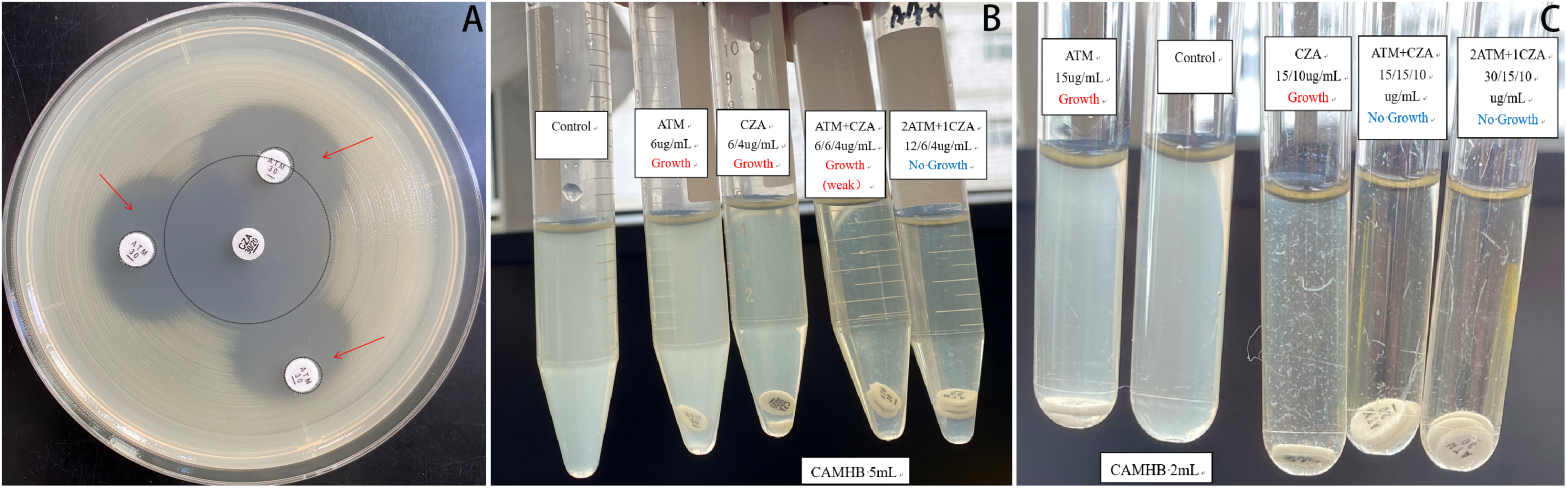
Combined antimicrobial susceptibility testing. **(A)** Double-disk synergy test: The CZA and ATM disks were placed on an agar plate inoculated with *S. maltophilia*, ensuring that their centers were positioned 10–20 mm apart. Following incubation, the morphological characteristics of the inhibition zones between the two disks were meticulously examined. A notably enlarged and more distinct zone of inhibition was observed at the interface compared to those produced by each drug alone, indicating a synergistic interaction between CZA and ATM. **(B, C)** Broth disk elution assay: The necessary materials were prepared, including CZA and ATM disks, cation-adjusted Mueller-Hinton broth (CAMHB), and sterile test tubes. Initially, the turbidity of the *S. maltophilia* isolate was adjusted to conform to a 0.5 McFarland standard. Subsequently, a 25µL aliquot of the standardized inoculum was added to 5 mL tubes containing Growth Control, ATM, CZA, and ATM + CZA (10 µL added to 2 mL tubes, respectively), achieving a final inoculum concentration of approximately 7.5 × 10^5^ CFU/mL. Using aseptic techniques, the ATM or CZA disk was carefully added to the tube. The tubes were incubated at 35 °C in ambient air for 20 hours. Purity was verified through Gram staining, and growth results were recorded the following day. Ultimately, various combinations of CZA and ATM were evaluated. The results indicated that the combination of 12 µg/mL ATM with 6/4 µg/mL CZA exhibited the minimum concentration required for complete sterilization. CZA, ceftazidime-avibactam; ATM, aztreonam.

On the 20th day in the SICU, a chest CT scan revealed small bilateral pleural effusions. The PaO_2_/FIO_2_ ratio was 220 mmHg, T was 36.8°C, WBC was elevated at 19.38 × 10^9^/L, N% was elevated at 90.5%, Procalcitonin (PCT) levels were markedly elevated at 33.64 ng/mL, and urine output was 2400mL/day. Blood cultures identified *S. maltophilia*, with susceptibility testing indicating sensitivity to minocycline, levofloxacin, and TMP-SMX (**Table 2**). The Cr was 142.9 μmol/L; therefore, TMP-SMX was not selected. Instead, oral minocycline 100 mg once daily was initiated. Three days later (on SICU Day 23), infection markers showed no significant improvement (details provided in **Table 3**), and serum creatinine (Cr) had increased to 197 μmol/L. The repeat blood culture remained positive for *S. maltophilia*. Based on these findings and the results of combined antimicrobial susceptibility testing (**Figure 1**), the clinical pharmacist and attending physicians collaboratively decided to discontinue minocycline following a thorough clinical reassessment The treatment regimen was subsequently modified to include CZA 1.25 g every 8 hours in combination with ATM 1 g every 8 hours, administered via synchronized intravenous infusions over a duration of 3 hours. After one week of combination therapy (day 29 in the SICU), the patient’s infection markers demonstrated substantial improvement, and *S. maltophilia* was isolated from sputum cultures. With Cr levels decreasing to 120.3 μmol/L (CrCl approximately 40 mL/min) and urine output recorded at 2500 mL/day, the regimen was adjusted to ATM 2 g every 8 hours and CZA 2.5 g intravenously every 8 hours, administered as synchronized 3-hour infusions. Additionally, lung imaging revealed no signs of active infection, the PaO_2_/FIO_2_ ratio improved to 451 mmHg, the endotracheal tube was removed, and high-flow oxygen therapy (50% FiO_2_ at 50 L/min) was initiated, achieving full SaO_2_. Blood cultures were repeated every 2 to 3 days. After 9 days of combination therapy with CZA and ATM, the blood culture became negative on day 31 of the patient’s admission to the SICU. On day 34 of SICU admission, with the patient’s condition stabilized and serum creatinine further decreased to 107.4 μmol/L, CZA and ATM were discontinued. The antibiotic regimen was de-escalated to cefoperazone-sulbactam, and the patient was subsequently transferred to the general ward. Due to right groin ulceration, wound debridement was performed, and the wound healed well with supportive care. The patient was eventually discharged one month later.

**Table 2:** Results of the antimicrobial susceptibility tests for the *S. maltophilia* strain.

Antimicrobial agent	Clinical categorization	Zone diameter, mm	Clinical breakpoints	Experimental method
Levofloxacin	S	25	13-17	disk diffusion
TMP-SMX	S	25	10-16	disk diffusion
Minocycline	S	26	20-26	disk diffusion

Antimicrobial susceptibility testing was performed by the standard disk diffusion method described in CLSI M02 [*1], according to the Clinical and Laboratory Standards Institute (CLSI) 2025 breakpoints [*2].

[*1] CLSI. Performance Standards for Antimicrobial Disk Susceptibility Tests. 14th ed. CLSI standard M02. Clinical and Laboratory Standards Institute; 2024.

[*2] CLSI. Performance Standards for Antimicrobial Susceptibility Testing. 35th ed. CLSI supplement M100. Clinical and Laboratory Standards Institute; 2025.

**Table 3:** Laboratory indicators associated with patient during the treatment of *S. maltophilia*.

Time	D19	D20	D23	D24	D29	D34
T °C	36.2	36.8	36.6	36.5	36.5	36
WBC 10^9/L	21.91	19.38	22.55	19.53	13.74	9.91
N %	93.9	90.5	86.8	87	82.3	78.7
PCT ng/mL	33.64		4.36	1.9		0.83
Cr umol/L	142.9			197	120.3	118.4

## Discussion

The Chinese Expert Consensus on *S. maltophilia* infections recommends TMP-SMX as the first-line agent (Zhou et al., 2013). However, since TMP-SMX is primarily excreted through the kidneys, impaired renal function can prolong its elimination half-life, potentially leading to systemic accumulation of both the parent drug and its metabolites. In this case, TMP-SMX was contraindicated due to the patient’s severely compromised renal function (CrCl≈27mL/min). Both the Chinese Expert Consensus and IDSA guidelines suggest tetracyclines—particularly minocycline—as alternative therapeutic options for patients with renal impairment. Nevertheless, minocycline’s high lipophilicity and large volume of distribution result in higher tissue concentrations compared to serum levels, which may limit its efficacy in treating bloodstream infections (Agwuh and MacGowan, 2006). The clinical team decided against the use of levofloxacin due to concerns regarding potential infection exacerbation, severe renal impairment, and an increased risk of neurotoxicity associated with its concomitant use with minocycline. Currently, there is no universally accepted optimal treatment regimen for *S. maltophilia* bacteremia in patients with renal dysfunction.

Recent studies on bloodstream infections have identified *S. maltophilia* as one of the pathogens associated with the highest mortality rates (Koh et al., 2025). Given this elderly patient’s ICU admission, along with multi-organ dysfunction and an immunocompromised status, prompt and effective antimicrobial therapy was essential to control the infection. This necessity prompted us to explore antimicrobial combination testing. The double-disk synergy test for CZA and ATM was performed according to the Chinese Expert Consensus on Combined Antimicrobial Susceptibility Testing (Li et al., 2023). The broth disk elution assay was conducted following the guidelines from the Clinical and Laboratory Standards Institute (CLSI) (Harris et al., 2023). In this study, *in vitro* experiments demonstrated significant inhibitory effects against *S. maltophilia* when CZA and ATM were combined at specific concentration ratios. This regimen has recently been recommended as a first-line treatment option for *S. maltophilia* in the 2024 IDSA guidelines for managing drug-resistant Gram-negative infections (Tamma et al., 2024). Both CZA and ATM are β-lactam antibiotics that exert time-dependent bactericidal activity by inhibiting bacterial cell wall synthesis. Unlike minocycline or TMP-SMX, these agents achieve higher serum concentrations compared to tissue concentrations, making them particularly suitable for the treatment of bloodstream infections (Mattie, 1994; Zhanel et al., 2013; Falcone et al., 2018; Wölfl-Duchek et al., 2025). For administration, we employed a simultaneous intravenous infusion using the ‘Y-site’ method, based on documented compatibility between CZA and ATM (O’Donnell et al, 2020). This strategy enhances bacterial clearance by prolonging the duration during which free drug concentrations exceed the minimum inhibitory concentration (MIC), especially during 3-hour infusions (Lodise et al., 2020). Extended or continuous infusions of β-lactams have been shown to improve clinical outcomes in critically ill patients (Hong et al., 2023; Abdul-Aziz et al., 2024), and therapeutic drug monitoring combined with pharmacokinetic/pharmacodynamic (PK/PD) analysis may further enhance treatment optimization.

In this case, the CZA-ATM combination was initiated with appropriate dose adjustments according to the patient’s renal function. After 12 days of therapy, the patient exhibited normalized infection markers, negative blood cultures, and improved renal function, indicating successful treatment.

*S. maltophilia* demonstrates reduced susceptibility to a wide range of antibiotics due to intrinsic resistance mechanisms, including low membrane permeability, chromosomally encoded multidrug efflux pumps, and the production of two inducible β-lactamases (L1 and L2), which confer resistance to most clinically used β-lactams (Sánchez, 2015). The L1 enzyme, classified as a metallo-β-lactamase (class B), hydrolyzes carbapenems and various β-lactams—excluding ATM—and remains unaffected by all currently available β-lactamase inhibitors (Crossman et al., 2008; Okazaki and Avison, 2008). In contrast, the L2 enzyme, categorized as a cephalosporinase (class A), mediates resistance to broad-spectrum cephalosporins and ATM but can be inhibited by commercial serine β-lactamase inhibitors such as clavulanate and avibactam (Mojica et al., 2017). These mechanistic insights provide a rationale for combining CZA with ATM to overcome the β-lactamase-mediated resistance of *S. maltophilia*.

Current clinical evidence regarding the use of CZA in combination with ATM for treating *S. maltophilia* infections remains limited. A. Diarra et al. reported a successful outcome in a patient with idiopathic medullary aplasia and multidrug-resistant *S. maltophilia* bloodstream infection treated with avibactam (AVI)-ATM combination therapy (Diarra et al., 2021). Similarly, Mojica et al. documented successful management of *S. maltophilia* bacteremia in a renal transplant recipient using CZA-ATM (Mojica et al., 2016). *In vitro* studies have further demonstrated that the ATM-AVI combination exhibits inhibitory activity against 97.8% of global *S. maltophilia* isolates collected from multiple medical centers (Sader et al., 2020). Another study evaluating ATM in combination with β-lactamase inhibitors against *S. maltophilia* found that AVI restored ATM susceptibility in 98% of ATM-resistant isolates (Biagi et al., 2020). Chinese *in vitro* combination studies revealed that avibactam restored ceftazidime (CAZ) and ATM activity in 48.48% (16/33) and 89.71% (61/68) of *S. maltophilia* isolates, respectively (Lin et al., 2021). Notably, some studies suggest that the ATM-amoxicillin-clavulanate combination may offer comparable efficacy to ATM-CZA against *S. maltophilia*, with potential cost advantages; however, this hypothesis requires further clinical validation (Emeraud et al., 2019).

Limitations and Future Perspectives: This case report contributes to the scarce real-world evidence regarding the use of the CZA-ATM combination in the treatment of *S. maltophilia* BSI. It is critical to recognize that our findings are derived from a single patient, limiting generalizability. As emphasized in multiple recent comprehensive reviews (Mojica et al., 2023; Carbonell et al, 2024; Geremia et al., 2025; Monardo et al., 2025), the management of *S. maltophilia* infections continues to pose significant therapeutic challenges, underscoring the urgent need for expanded clinical data, standardized susceptibility testing methodologies, and the development of novel antimicrobial agents to inform evidence-based treatment strategies for these life-threatening infections.

## Conclusions

This case involved an elderly patient with a bloodstream infection caused by *S. maltophilia*, which was complicated by multi-organ failure. Based on relevant clinical evidence and the results of antimicrobial combination testing, a treatment regimen consisting of CZA combined with ATM was selected. This therapy demonstrated confirmed efficacy and safety, providing a potential reference for clinical practice. Although further large-scale clinical studies are warranted, ATM-AVI may represent a valuable therapeutic option for managing *S. maltophilia* infections.

## Data Availability

The original contributions presented in the study are included in the article/supplementary material, Further inquiries can be directed to the corresponding authors.

## References

[B1] Abdul-Aziz M. H. Hammond N. E. Brett S. J. Cotta M. O. De Waele J. J. Devaux A.. (2024). Prolonged vs intermittent infusions of β-lactam antibiotics in adults with sepsis or septic shock: A systematic review and meta-analysis. Jama. 332, 638–648. doi: 10.1001/jama.2024.9803, PMID: 38864162 PMC11170459

[B2] Agwuh K. N. MacGowan A. (2006). Pharmacokinetics and pharmacodynamics of the tetracyclines including glycylcyclines. J. antimicrobial chemotherapy. 58, 256–265. doi: 10.1093/jac/dkl224, PMID: 16816396

[B3] Biagi M. Lamm D. Meyer K. Vialichka A. Jurkovic M. Patel S.. (2020). Activity of Aztreonam in Combination with Avibactam, Clavulanate, Relebactam, and Vaborbactam against Multidrug-Resistant Stenotrophomonas maltophilia. Antimicrobial Agents chemotherapy. 64(12):e00297-20. doi: 10.1128/AAC.00297-20, PMID: 32928733 PMC7674038

[B4] Bostanghadiri N. Sholeh M. Navidifar T. Dadgar-Zankbar L. Elahi Z. van Belkum A.. (2024). Global mapping of antibiotic resistance rates among clinical isolates of Stenotrophomonas maltophilia: a systematic review and meta-analysis. Ann. Clin. Microbiol. antimicrobials. 23, 26. doi: 10.1186/s12941-024-00685-4, PMID: 38504262 PMC10953290

[B5] Brooke J. S. (2012). Stenotrophomonas maltophilia: an emerging global opportunistic pathogen. Clin. Microbiol. Rev. 25, 2–41. doi: 10.1128/CMR.00019-11, PMID: 22232370 PMC3255966

[B6] Carbonell N. Oltra M. R Clari M. (2024). Stenotrophomonas maltophilia: the landscape in critically ill patients and optimizing management approaches. Antibiotics (Basel Switzerland). 13(7):577. doi: 10.3390/antibiotics13070577, PMID: 39061259 PMC11273807

[B7] Crossman L. C. Gould V. C. Dow J. M. Vernikos G. S. Okazaki A. Sebaihia M.. (2008). The complete genome, comparative and functional analysis of Stenotrophomonas maltophilia reveals an organism heavily shielded by drug resistance determinants. Genome Biol. 9, R74. doi: 10.1186/gb-2008-9-4-r74, PMID: 18419807 PMC2643945

[B8] Diarra A. Pascal L. Carpentier B. Baclet N. Cabaret P. Georgel A. F.. (2021). Successful use of avibactam and aztreonam combination for a multiresistant Stenotrophomonas maltophilia bloodstream infection in a patient with idiopathic medullary aplasia. Infect. Dis. now. 51, 637–638. doi: 10.1016/j.idnow.2021.01.014, PMID: 33870895

[B9] Ding L. Guo Y. Wu S. Yang Y. Yin D. Han R.. (2024). CHINET China bacterial resistance monitoring results, 2024 Edition. Institute of Antibiotics, Huashan Hospital, Fudan University, Shanghai, China. 2024 Edition.

[B10] Emeraud C. Escaut L. Boucly A. Fortineau N. Bonnin R. A. Naas T and Dortet L. (2019). Aztreonam plus clavulanate, tazobactam, or avibactam for treatment of infections caused by metallo-β-lactamase-producing gram-negative bacteria. Antimicrobial Agents chemotherapy. 63(5):e00010-19. doi: 10.1128/AAC.00010-19, PMID: 30858212 PMC6496057

[B11] Falcone M. Viale P. Tiseo G and Pai M. (2018). Pharmacokinetic drug evaluation of avibactam + ceftazidime for the treatment of hospital-acquired pneumonia. Expert Opin. Drug Metab. toxicology. 14, 331–340. doi: 10.1080/17425255.2018.1434142, PMID: 29373935

[B12] Fihman V. Le Monnier A. Corvec S. Jaureguy F. Tankovic J. Jacquier H.. (2012). Stenotrophomonas maltophilia–the most worrisome threat among unusual non-fermentative gram-negative bacilli from hospitalized patients: a prospective multicenter study. J. infection. 64, 391–398. doi: 10.1016/j.jinf.2012.01.001, PMID: 22245400

[B13] Geremia N. Marino A. De Vito A. Giovagnorio F. Stracquadanio S. Colpani A.. (2025). Rare or unusual non-fermenting gram-negative bacteria: therapeutic approach and antibiotic treatment options. Antibiotics (Basel Switzerland). 14(3):306. doi: 10.3390/antibiotics14030306, PMID: 40149115 PMC11939765

[B14] Gil-Gil T. Martínez JL. Blanco P. (2020). Mechanisms of antimicrobial resistance in Stenotrophomonas maltophilia: a review of current knowledge. Expert Rev. anti-infective Ther. 18, 335–347. doi: 10.1080/14787210.2020.1730178, PMID: 32052662

[B15] Harris H. Tao L. Jacobs E. B. Bergman Y. Adebayo A. Tekle T.. (2023). Multicenter evaluation of an MIC-based aztreonam and ceftazidime-avibactam broth disk elution test. J. Clin. Microbiol. 61, e0164722. doi: 10.1128/jcm.01647-22, PMID: 37070979 PMC10204635

[B16] Hong L. T. Downes K. J. FakhriRavari A. Abdul-Mutakabbir J. C. Kuti J. L. Jorgensen S.. (2023). International consensus recommendations for the use of prolonged-infusion beta-lactam antibiotics: Endorsed by the American College of Clinical Pharmacy, British Society for Antimicrobial Chemotherapy, Cystic Fibrosis Foundation, European Society of Clinical Microbiology and Infectious Diseases, Infectious Diseases Society of America, Society of Critical Care Medicine, and Society of Infectious Diseases Pharmacists. Pharmacotherapy. 43, 740–777. doi: 10.1002/phar.2842, PMID: 37615245

[B17] Huang C. Lin L. Kuo S. (2024). Risk factors for mortality in Stenotrophomonas maltophilia bacteremia - a meta-analysis. Infect. Dis. (London England). 56, 335–347. doi: 10.1080/23744235.2024.2324365, PMID: 38436567

[B18] Kanchanasuwan S. Rongmuang J. Siripaitoon P. Kositpantawong N. Charoenmak B. Hortiwakul T.. (2022). Clinical characteristics, outcomes, and risk factors for mortality in patients with stenotrophomonas maltophilia bacteremia. J. Clin. Med. 11(11):3085. doi: 10.3390/jcm11113085, PMID: 35683471 PMC9181236

[B19] Koh M. C. Y. Ngiam J. N. Chew K. L. Smitasin N. Lum LH and Allen D. M. (2025). Clinical presentation and outcomes of bloodstream infection with intrinsically carbapenem-resistant non-fermenting gram-negative organisms: Stenotrophomonas maltophilia, Elizabethkingia spp. and Chryseobacterium spp. in Singapore, from 2012 to 2024. BMC Infect. diseases. 25, 273. doi: 10.1186/s12879-025-10636-9, PMID: 40000958 PMC11863829

[B20] Li D. Baiyi C. Min L. Yuxing N. Bin S. Danhong S.. (2023). Expert consensus on antimicrobial synergy testing and reporting of carbapenem-resistant Gram-negative bacteria. Chin. J. Infection Chemotherapy. 23(1):80–90. doi: 10.16718/j.1009-7708.2023.01.013

[B21] Lin Q. Zou H. Chen X. Wu M. Ma D. Yu H.. (2021). Avibactam potentiated the activity of both ceftazidime and aztreonam against S. maltophilia Clin. isolates vitro. BMC Microbiol. 21, 60. doi: 10.1186/s12866-021-02108-2, PMID: 33618662 PMC7901100

[B22] Lodise T. P. Smith N. M. O’Donnell N. Eakin A. E. Holden P. N. Boissonneault K. R.. (2020). Determining the optimal dosing of a novel combination regimen of ceftazidime/avibactam with aztreonam against NDM-1-producing Enterobacteriaceae using a hollow-fiber infection model. J. antimicrobial chemotherapy. 75, 2622–2632. doi: 10.1093/jac/dkaa197, PMID: 32464664 PMC8444334

[B23] Majumdar R. Karthikeyan H. Senthilnathan V. Sugumar S. (2022). Review on Stenotrophomonas maltophilia: An Emerging Multidrug- resistant Opportunistic Pathogen. Recent patents Biotechnol. 16, 329–354. doi: 10.2174/1872208316666220512121205, PMID: 35549857

[B24] Mattie H. (1994). Clinical pharmacokinetics of aztreonam. update. Clin. pharmacokinetics. 26, 99–106. doi: 10.2165/00003088-199426020-00003, PMID: 8162661

[B25] Mojica M. F. Bonomo R. A. van Duin D. (2023). Treatment approaches for severe Stenotrophomonas maltophilia infections. Curr. Opin. Infect. diseases. 36, 572–584. doi: 10.1097/QCO.0000000000000975, PMID: 37846568

[B26] Mojica M. F. Ouellette C. P. Leber A. Becknell M. B. Ardura M. I. Perez F.. (2016). Successful treatment of bloodstream infection due to metallo-β-lactamase-producing stenotrophomonas maltophilia in a renal transplant patient. Antimicrobial Agents chemotherapy. 60, 5130–5134. doi: 10.1128/AAC.00264-16, PMID: 27551008 PMC4997835

[B27] Mojica M. F. Papp-Wallace K. M. Taracila M. A. Barnes M. D. Rutter J. D. Jacobs M. R.. (2017). Avibactam restores the susceptibility of clinical isolates of stenotrophomonas maltophilia to aztreonam. Antimicrobial Agents chemotherapy. 61(10):e00777-17. doi: 10.1128/AAC.00777-17, PMID: 28784669 PMC5610502

[B28] Monardo R. Mojica M. F. Ripa M. Aitken S. L. Bonomo R. A. van Duin D. (2025). How do I manage a patient with Stenotrophomonas maltophilia infection? Clin. Microbiol. infection: Off. Publ. Eur. Soc. Clin. Microbiol. Infect. Diseases. 31, 1291–1297. doi: 10.1016/j.cmi.2025.04.031, PMID: 40339792

[B29] O’Donnell J. N. Xu A. Lodise T. P. (2020). Intravenous compatibility of ceftazidime-avibactam and aztreonam using simulated and actual Y-site administration. Clin. Ther. 42, 1580–1586.e2. doi: 10.1016/j.clinthera.2020.06.005, PMID: 32684326 PMC8428177

[B30] Okazaki A. Avison M. B. (2008). Induction of L1 and L2 beta-lactamase production in Stenotrophomonas maltophilia is dependent on an AmpR-type regulator. Antimicrobial Agents chemotherapy. 52, 1525–1528. doi: 10.1128/AAC.01485-07, PMID: 18212097 PMC2292540

[B31] Sader H. S. Duncan L. R. Arends S. J. R. (2020). Carvalhaes CG and Castanheira M. Antimicrobial Activity of Aztreonam-Avibactam and Comparator Agents When Tested against a Large Collection of Contemporary Stenotrophomonas maltophilia Isolates from Medical Centers Worldwide. Antimicrobial Agents chemotherapy. 64(11):e01433-20. doi: 10.1128/AAC.01433-20, PMID: 32900683 PMC7577171

[B32] Sánchez M. B. (2015). Antibiotic resistance in the opportunistic pathogen Stenotrophomonas maltophilia. Front. Microbiol. 6, 658. doi: 10.3389/fmicb.2015.00658, PMID: 26175724 PMC4485184

[B33] Tamma P. D. Heil E. L. Justo J. A. Mathers A. J. Satlin MJ and Bonomo R. A. (2024). Infectious diseases society of america 2024 guidance on the treatment of antimicrobial-resistant gram-negative infections. Clin. Infect. diseases: an Off. Publ. Infect. Dis. Soc. America. ciae403. doi: 10.1093/cid/ciae403, PMID: 39108079

[B34] Wölfl-Duchek M. van Os W. Al Jalali V. Rabl U. Wohlrab P. Bauer M.. (2025). Cerebrospinal fluid concentrations of ceftaroline and ceftazidime/avibactam in healthy volunteers: Pharmacokinetics and probability of target attainment. Int. J. antimicrobial agents. 66, 107512. doi: 10.1016/j.ijantimicag.2025.107512, PMID: 40239748

[B35] Zhanel G. G. Lawson C. D. Adam H. Schweizer F. Zelenitsky S. Lagacé-Wiens P. R.. (2013). Ceftazidime-avibactam: a novel cephalosporin/β-lactamase inhibitor combination. Drugs. 73, 159–177. doi: 10.1007/s40265-013-0013-7, PMID: 23371303

[B36] Zhou H. Li G. Zhuo C. Yang Y. Shi Y. Chen B.. (2013). Chinese expert consensus on the diagnosis, treatment, and prevention of stenotrophomonas maltophilia infections. Chin. Med. J. 93(16):1203–121. doi: 10.3760/cma.j.issn.0376-2491.2013.16.002

